# Interplay of Magnetic Interactions and Active Movements in the Formation of Magnetosome Chains

**DOI:** 10.1371/journal.pone.0033562

**Published:** 2012-03-19

**Authors:** Stefan Klumpp, Damien Faivre

**Affiliations:** 1 Department Theory and Bio-Systems, Max Planck Institute of Colloids and Interfaces, Potsdam, Germany; 2 Department Biomaterials, Max Planck Institute of Colloids and Interfaces, Potsdam, Germany; George Mason University, United States of America

## Abstract

Magnetotactic bacteria assemble chains of magnetosomes, organelles that contain magnetic nano-crystals. A number of genetic factors involved in the controlled biomineralization of these crystals and the assembly of magnetosome chains have been identified in recent years, but how the specific biological regulation is coordinated with general physical processes such as diffusion and magnetic interactions remains unresolved. Here, these questions are addressed by simulations of different scenarios for magnetosome chain formation, in which various physical processes and interactions are either switched on or off. The simulation results indicate that purely physical processes of magnetosome diffusion, guided by their magnetic interactions, are not sufficient for the robust chain formation observed experimentally and suggest that biologically encoded active movements of magnetosomes may be required. Not surprisingly, the chain pattern is most resembling experimental results when both magnetic interactions and active movement are coordinated. We estimate that the force such active transport has to generate is compatible with forces generated by the polymerization or depolymerization of cytoskeletal filaments. The simulations suggest that the pleiotropic phenotypes of *mamK* deletion strains may be due to a defect in active motility of magnetosomes and that crystal formation in magneteosome vesicles is coupled to the activation of their active motility in *M. gryphiswaldense*, but not in *M. magneticum*.

## Introduction

Magnetotactic bacteria have the ability to orient and navigate in the magnetic field of the Earth with the help of special magnetic organelles called magnetosomes [1]. Magnetosomes are membrane-enclosed nano-sized crystals of a magnetic mineral, typically magnetite (Fe_3_O_4_), which are assembled in chains along the cell axis. The generated magnetic dipole moment is large enough so that its interaction energy with the magnetic field of the Earth overcomes thermal fluctuations and allows cells to align and to swim along field lines. The latter behavior is known as magnetotaxis [2] and is believed to direct the bacteria towards environmental conditions favorable for growth [3]. In recent years, magnetotactic bacteria have been studied extensively from a variety of perspectives. Originally, magnetotactic bacteria have mostly been investigated in the context of environmental microbiology for their diversity in both phylogeny and habitats [4], [5], [6] and in geosciences, as fossil magnetosomes contribute to the magnetism of sediments [7]. More recently, they have become model systems for biomineralization because of their specific crystal morphologies, their chemical purity and their quasi-one dimensional organization [8], [9]. In biotechnology, the use of their magnetic properties for bioremediation and other applications is also currently explored [10], [11]. Finally, new imaging techniques for bacterial cell biology provide tools to study in detail the dynamics and the spatial organization of the interior of these cells, which allows to study magnetosomes as a model system for the formation of organelles in bacteria [12]. From a biophysics point of view, this process also provides an opportunity to address the interplay of physical processes with biological regulation mechanisms.

The formation of magnetosomes is a complex process that consists of the controlled biomineralization of magnetite in pre-existing vesicles and the synergic assembly of magnetosomes into chains [9]. The detailed mechanism of this process remains unknown, but it is expected to involve specific biological control mechanisms as well as generic physical processes and interactions such as the diffusion of magnetosomes in the cell and the magnetic interactions between the nanocrystals. Several molecular players involved in the formation and assembly of magnetosomes have indeed already been identified. These include the cytoskeletal proteins MamK and MamJ that play a role in the assembly of magnetosome chains [13], [14], [15] and several proteins that affect the size of the crystals [16]. Studies of the formation of magnetosomes in iron-starved cells, mostly of *Magnetospirilla* strains, also provide some constraints on the dynamics of the processes. It was for example shown that about 6 hours were necessary for the magnetosomes to grow and reach a mature size, and to assemble into the typical chain arrangement with narrowly spaced neighbor-crystallites [17], [18], [19]. Moreover, it was hypothesized based on FMR spectroscopy that the crystals first growing over the critical superparamagnetic to stable single domain size threshold (∼25 nm), act as ‘magnetosome docks’ and play a decisive role for the stabilization of the magnetic dipole and thus for chain formation [20].

However, a number of key questions have remained open. In general, how are physical interactions coordinated with specific biological (transport) mechanisms? And more specifically, what is the role of magnetic interactions in the formation of the magnetosome chain? Are they, together with diffusive movements of the magnetosomes, sufficient for the formation of a chain? Likewise, what are the roles of specific, genetically encoded, factors? The discovery of the MamK and MamJ proteins has pointed towards a role for cytoskeletal structures in the process of magnetosome chain formation: MamK forms (or is at least a crucial part of) a filamentous structure, the magnetosome filament, that extends along the cell axis [13]. Magnetosomes are aligned along that filament [13], [21] and linked to it through the MamJ protein [14]. Contacts of magnetosomes with cytoskeletal filaments are clearly required, as freely diffusing magnetosomes do not form linear assemblies, but rather collapse into unstructured clusters [22]. A minimal model for the role of the cytoskeleton in this context is therefore that the magnetosome filament provides a structural scaffold for the formation of a linear assembly of magnetosomes. However, beyond such purely structural role, cytoskeletal structures could also have a more dynamic function and be involved in directed transport, delivering magnetosomes to the site of the formation of a magnetosome chain [8]. The minimal model of a purely structural role of the magnetosome filament is indeed challenged by recent observations that a *mamK* deletion mutant forms (short) magnetosome chains, but is defective in assembling a single chain as well as in the midcell localization of magnetosomes [15]. A more dynamic role of the magnetosome filament is also consistent with observations that MamK filaments are dynamic and depolymerize in an active, ATP-dependent fashion [23], [24], [25].

Here, we use computer simulations of the formation of magnetosomes to address these questions. We have developed a computer model that integrates generic physical processes with specific biological functions. Specifically, the model describes the nucleation and growth of magnetite crystals, the diffusive and active transport of magnetosomes, and their magnetic interactions. We use this model to simulate several scenarios of the dynamics of magnetosome formation and assembly. Simulating these scenarios allows us to study ‘in-silico mutants’ that have various physical processes and/or interactions turned on or off, in order to elucidate the roles of the different dynamic processes. In some cases, it may be possible to obtain corresponding experimental mutants by genetic modification, although it is currently not known which genes encode proteins with the predicted functions. In other cases such as those with magnetic interactions turned off, it is very unlikely that such mutants can ever be generated experimentally at all. The simulations therefore allow us to elucidate the roles of different molecular players, dynamical processes and physical interactions in a complementary manner to current experimental studies of magnetosome formation and assembly.

Our simulations indicate that the magnetic interactions between magnetosomes are not sufficient for the reliable formation of a magnetosome chain and suggest that chain formation is driven by the interplay of active transport of magnetosomes and magnetic interactions: Active movements are likely to be the main driving force for forming and centering the magnetosome chain, while magnetic interactions have a role in stabilizing the chain. We estimate the force required to power active transport and find it to be easily accessible by the polymerization or depolymerization of cytoskeletal filaments. Our results also suggest to interpret recent observations in a *mamK* deletion mutant of *M. gryphiswaldense* [15] as the results of a defect in active transport.

## Results

### Model for magnetosome chain formation

**Figure 1:**
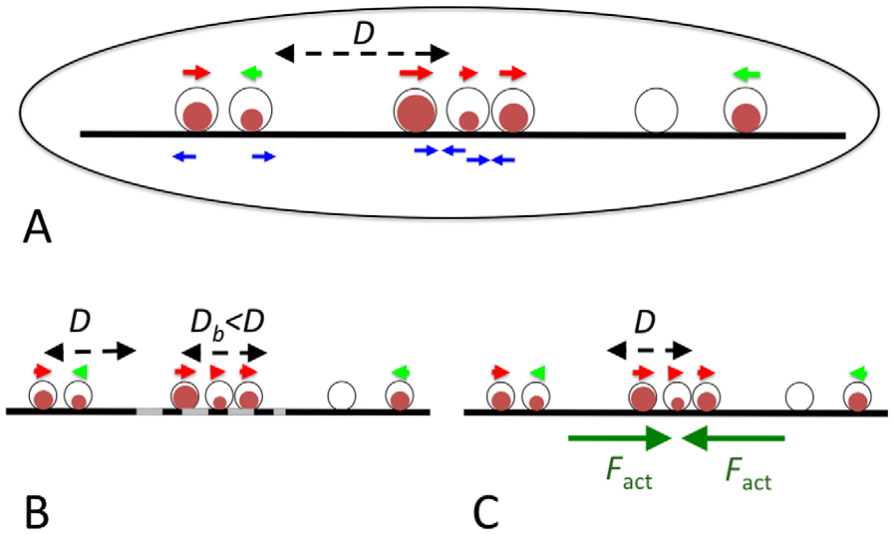
Model for magnetosome dynamics and scenarios of chain formation. (A) The basic scenario: Magnetosomes are described by their position, the volume of the magnetite crystal they contain (brown circles) and the direction of their magnetic momentum (indicated by small red and green arrows). They move diffusively with diffusion coefficient *D* in one dimension, constrained by the cytoskeletal magnetosome filament along the cell axis, and interact through magnetic dipole-dipole interactions (A), which may either be attractive or repulsive (blue arrows). In other scenarios we simulate, the dynamics is modified by introducing a binding zone (dashed grey), in which diffusion is reduced by binding to the cytoskeleton (B), or active movement towards the center of the cell (C).

To study the driving forces of magnetosome chain formation, we developed a stochastic model describing the dynamics of magnetosomes in a bacterial cell (Figure 1). The model describes the nucleation and growth of the magnetite crystals in magnetosome vesicles, their magnetic moment, and the movements of the magnetosomes in the cells. Each magnetosome is described by its position in the cell (*x_i_*), the size of the crystal it contains (*V_i_*) and the orientation of its magnetic moment (*m_i_*). The dynamics of these three degrees of freedom is simulated using a combined Langevin dynamics-Monte Carlo approach (see Methods). In these simulations, we model the spatial degrees of freedom of the magnetosomes as one-dimensional movements to mimic their arrangement along the magnetosome filament, a cytoskeletal structure based on the MamK protein [13].

The values of most parameters used in the model are known or can be estimated directly from experimental data for *M. gryphiswaldense*; these parameter values are summarized in Table 1. The parameters of crystal growth are fixed by comparison with experimental data (see below). However, a key parameter that remains unknown is the mobility of the magnetosomes in the cell, characterized by the diffusion coefficient *D*. To our knowledge, the only experiment so far that studied diffusion of magnetosomes in vivo, was a Mössbauer spectroscopy study that reported a diffusion coefficient of ∼1 µm^2^/s for diffusive movements on very short time and length scales [26]. We take this value as an upper bound for the effective diffusion coefficient over larger scales. The diffusion is indeed expected to be smaller due to cytoplasmic barriers such as contacts to cytoskeletal elements or the cell membrane, which effectively increase the viscosity. Another estimate of the diffusion coefficient can be obtained from the measured diffusion coefficients of proteins in bacterial cytoplasm, which are also in the µm^2^/s range [27], [28]. As magnetosomes are larger by about one order of magnitude, their diffusion coefficient can be estimated to be ∼0.1 µm^2^/s, but this value is likely further reduced due to transient binding to cytoskeletal structures. Because of this uncertainty, the value of the diffusion coefficient was varied over several orders of magnitude in the simulations.

**Table 1:** Parameters of the simulation.

Parameter	Symbol	Value	Notes
Cell length	*L*	4 µm	
Number of magnetosome vesicles	*N*	30	[18]
Size of magnetosome vesicles and maximal crystal size	*R*_max_	25 nm	[20]
Nucleation rate	ν	10^−4^ s^−1^ = 0.36 hr^−1^	Figure 2A
Crystal growth rate	*v*_gr_	0.7 nm^3^/s	Figure 2B
Magnetization of magnetite	μ	4.8·10^−4^ A/nm	[1]
Critical radius for transition from superparamagnetic to stable single domain state	*R*_crit_	15 nm	[46], [48]
Coercive field	*B*_coerc_	12 mT	[19]
Length of short-range repulsive interaction, allowed overlap	d	2 nm	
Short-range repulsive force	*F*_rep_	0.001 pN	
Magnetosome diffusion coefficient	*D*	1–10^5^ nm^2^/s	see Text
Probability for exchange of an empty vesicle and a crystal-containing magnetosome	*p*_sw_	0.1	see Methods
Length of binding zone	*L*_b_	0.5 or 1 mm	
Fold-reduction of mobility in the binding zone	*D*_b_/*D*	0.01	
Active force	*F*_act_	0.01–1 pN	
Simulation time step	Δ*t*	0.01 s	for D<10^4^ nm^2^/s
		0.001 s	for D≥10^4^ nm^2^/s

Using this model, we simulated several different scenarios (‘in silico mutants’) for magnetosome chain assembly, where different physical processes are either switched on or off (Figure 1). Throughout this work, we focus on the de novo formation of a magnetosome chain, which has been studied extensively in iron-starved non-growing *M. gryphiswaldense* cells that are shifted into a medium containing iron to induce the formation of magnetosomes [14], [15], [17], [18], [20], [29].

**Figure 2:**
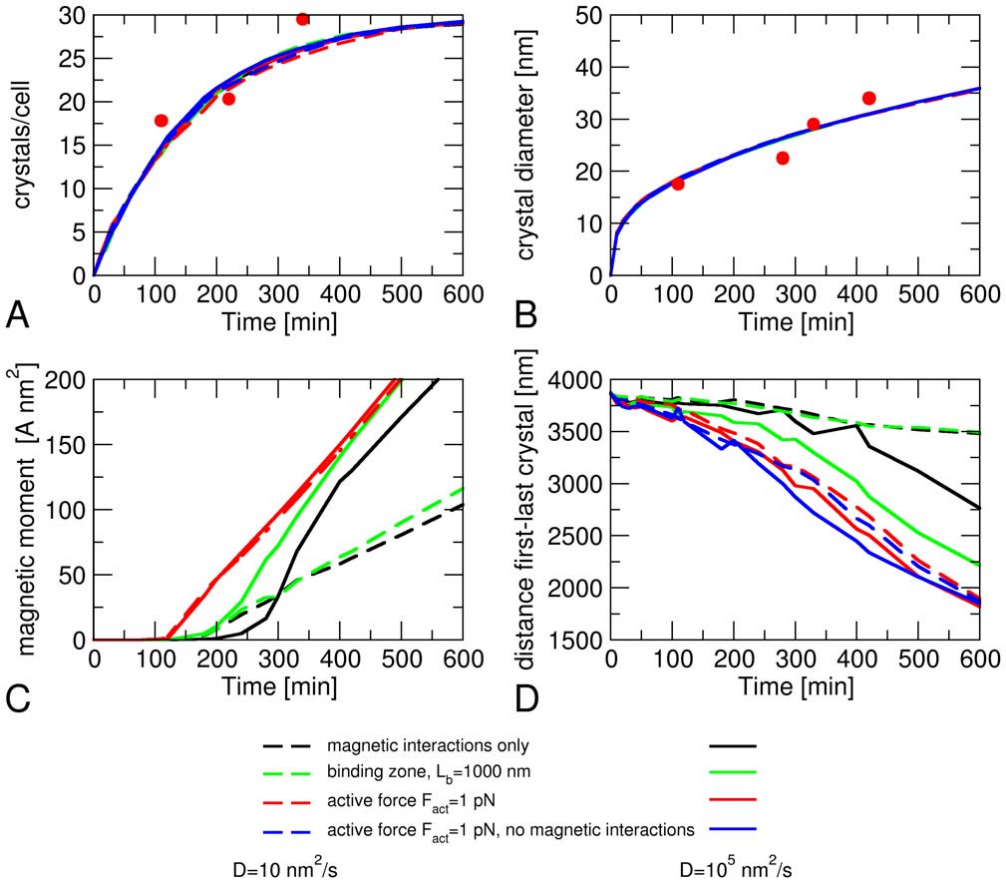
Simulated dynamics of magnetosome formation. (A) number of crystals per cell, (B) average crystal diameter, (C) average magnetic moment of a cell, and (D) average distance *d*_l-r_ between the leftmost and rightmost magnetosome as functions of time after the induction of magnetosome formation. The colors of the curves indicate the interactions and transport mechanisms included in the simulations, solid lines are for high magnetosome mobility (*D* = 0.01 mm^2^/s), dashed lines for low mobility (*D* = 10 nm^2^/s). The experimental data points are from Refs. [18] (A) and [20] (B).

In our model, magnetite crystals are nucleated stochastically in the magnetosome vesicles (with a rate ν) and, once nucleated, their volume grows deterministically with velocity *v*_gr_, until they reach the maximal size. By the design of the model, the growth of the magnetite crystals is independent of the spatial dynamics of the magnetosomes. This means that the observed number and size of the crystals do not depend on the value of the magnetosome diffusion coefficient or the presence or absence of active movements or other factors that influence the spatial dynamics of the magnetosomes (Figure 2A and 2B). The parameters of nucleation and growth (ν and *v*_gr_) can therefore be chosen such that they are the same in all our simulations for different dynamics. We have fixed these parameters by matching the time-dependence of the number and average radius of the crystals to experimental data from Refs. [18], [20] (Figure 2A and 2B).

### Case study 1: magnetism on and active movement off

We first asked whether magnetic interactions alone are sufficient to drive the assembly of magnetosome chains and therefore simulated the dynamics of magnetosomes in the absence of active movements. We will argue below that this case may correspond to the situation in a *mamK* deletion mutant. As the mobility of the magnetosomes is unknown, we have obtained data for a wide range of diffusion coefficients. Figure 2 shows simulation data for a high and a low value of the diffusion coefficient (black lines). In contrast to the growth of the crystals (Figure 2A and 2B), the average magnetization of the cells depends on the magnetosome mobility (Figure 2C), presumably because fast movements drive the crystals into close proximity where equal orientation of their magnetic moments is strongly favored. Figure 2D shows that magnetic interactions indeed result in attraction between the magnetosomes, as the average distance *d*_l-r_ between the leftmost and rightmost magnetosome decreases with time. For magnetosomes with low mobility (dashed black line in Figure 2), this decrease is, however, very weak. A diffusion coefficient of at least 100 nm^2^/s is required to see a pronounced decrease of *d*_l-r_ over a few hours. But even for high magnetosome mobility (solid black line in Figure 2D), the minimal distance reached over the relevant time scale (5–10 hours) remains substantially larger (>2700 nm) than the size of a single magnetosome chain with closely packed magnetosomes (1450 nm for 30 magnetosomes).

**Figure 3:**
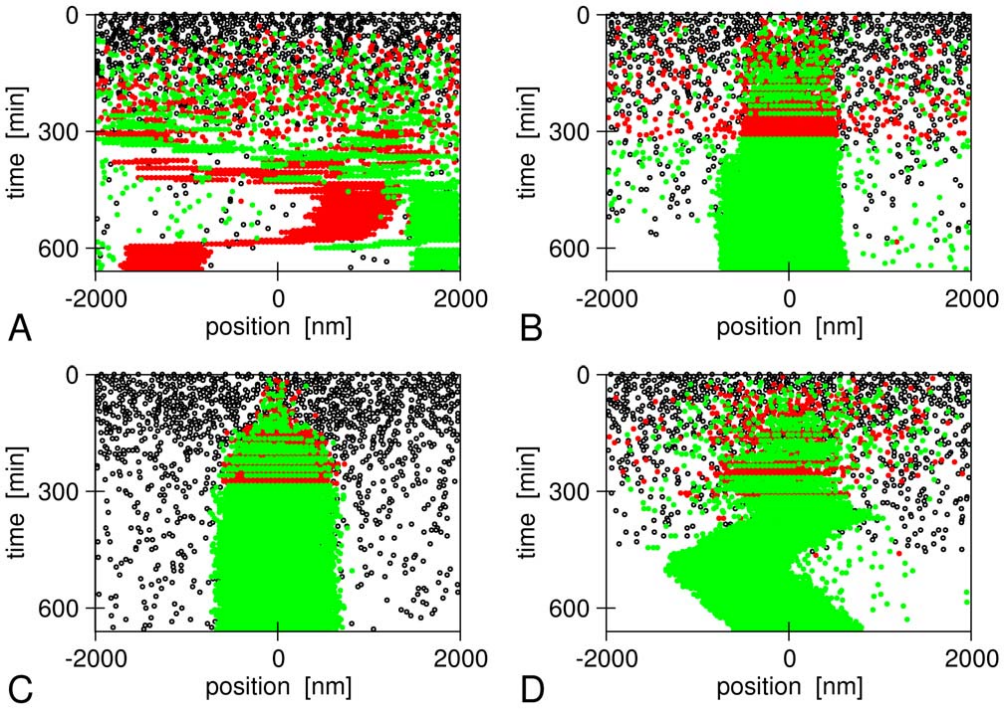
Example time traces of magnetosome formation in our simulations. (A) Magnetic interactions and diffusion only, (B) binding zone in the cell center (*L*_b_ = 1000 nm), (C) and (D) active transport to the cell center with an active force *F*_act_ = 1 pN (C) and 0.01 pN (D). In all panels, black dots indicate empty magnetosome vesicles, green and red points indicate magnetosomes containing a crystal with plus or minus orientation of its magnetic moment. In all panels, the magnetosome mobility is given by *D* = 10^5^ nm^2^/s.

**Figure 4:**
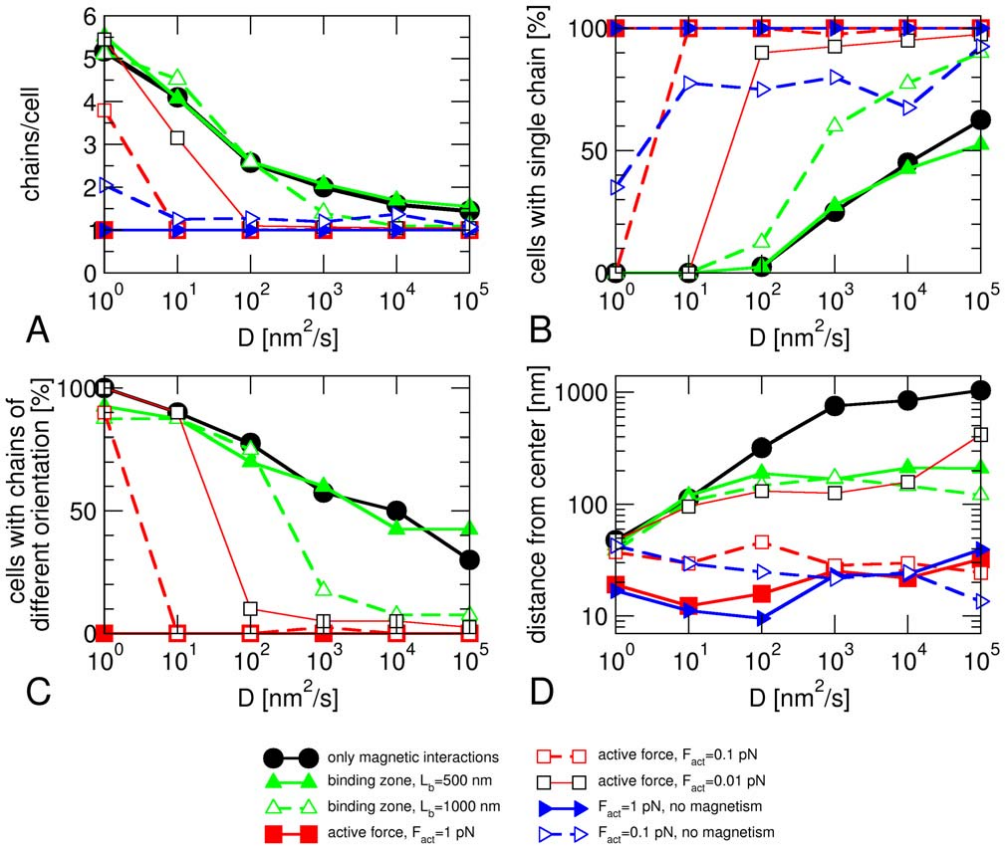
Analysis of the structures formed after 10 hours for different scenarios of magnetosome formation. (A) average number of chains per cell, (B) fraction of cell with a single chain, (C) fraction of cells with chains that have opposite orientations of their magnetic moment, (D) average distance of the center of mass of the magnetosomes from the cell center, plotted as functions of the magnetosome mobility, which is characterized by the diffusion coefficient *D*. For each scenario and each value of *D*, 40 cells were simulated and analyzed.

An inspection of the time traces of magnetosomes in a cell indicates that a large fraction of cells form several shorter chains rather than a single long one (Figure 3A). We thus determined the average number of chains per cell (Figure 4A) and the fraction of cells that have formed a single chain (Figure 4B) after 10 hours to quantify this observation. For this analysis, we counted two magnetosomes to be in the same chain if their edge-to-edge distance is less than 1.5 R_max_ and if their magnetic moments have the same orientation. Moreover, only assemblies consisting of at least three magnetosomes were taken into account and considered as chains. Figure 4A shows that the average number of chains per cell decreases with increasing magnetosome mobility, but that it remains considerably larger than 1 for all values of the diffusion coefficient we simulated (black circles). Likewise, the fraction of cells with a single chain grows with increasing magnetosome mobility, but even for the highest mobility (*D* = 10^5^ nm^2^/s) we find a substantial fraction with more than one chain (38% of the simulated cells). In addition, our simulations indicate that cells forming more than 1 chain typically have chains with oppositely oriented magnetic moments (Figure 4C). Finally, we have determined the average distance of the center of mass of the magnetosomes from the center of the cell (Figure 4D) and found that magnetosomes are not well-centered in these simulations, in particular for high magnetosome mobility. The low average distances from the center observed for low magnetosome mobility reflect the rather random location of multiple magnetosome chains in a cell rather than the centering of a single chain.

These observations show that diffusive movements of magnetosomes in the force field generated by their magnetic interactions may lead to the formation of a single magnetosome chain, provided that magnetosomes are sufficiently mobile. However, without additional directional clues, the chain will not be positioned properly in the center of the cell. In fact, if a single chain is formed, we often observe it to move diffusively through the whole cell. Furthermore, even with high mobility of magnetosomes, the formation of a chain is not very reliable and there is a substantial fraction of cells that form multiple chains instead of a single chain. We therefore conclude that diffusive magnetosome movements and magnetic interactions alone are not sufficient for the robust formation of a magnetosome chain.

### Case study 2: magnetism on and binding zone on

We next asked how the diffusive dynamics of magnetosomes had to be amended to ensure the formation of a single chain in the center of the cell. We have first tested the possibility that binding sites for magnetosomes in the cell center serve as a nucleus for the formation of a single chain. Such hypothetical binding sites could, for example, be located on cytoskeletal structures. Whether such sites exist and what molecular players could be involved is outside the scope of the current modeling. Likewise, how such sites could be positioned in the center of the cell is also not known. At the moment, it can only be speculated that a mechanism for positioning any object in the center of the cell might be similar to mechanisms known from other bacteria for positioning of the FtsZ ring and thus the cell division plane such as the Min system in *E.coli* [30], [31] or the MipZ system from *Caulobacter*, which is conserved among the α-proteobacteria (that include the *Magnetospirilla*) [32]. Assuming that such a mechanism is in place, binding sites for the magnetosomes were implemented by a reduced diffusion coefficient in an interval of length *L_b_* around the cell center. As the cells cannot be expected to ‘predict’ the exact size of the magnetosome chain that is yet to be formed, this length should be shorter than the final length of the magnetosome chain, i.e. *L_b_*<2*N R*_max_. We have performed simulations for various values of this length and various fold-reductions of the diffusion coefficient. Figure 2 includes data for a length *L*_b_ of 1000 nm (corresponding to binding sites for 20 densely packed magnetosomes) and a 100-fold lower diffusion coefficient in this region as compared to outside this region. The latter reduction may be interpreted as a magnetosome being immobilized by the binding sites during 99 percent of the time. For low magnetosome mobility, we see little difference compared to the case without such binding sites (compare the green and black dashed lines in Figure 2C and 2D), but for highly mobile magnetosomes, the magnetization increases slightly faster in the presence of such a mechanism than in its absence (Figure 2C) and the average distance *d*_l-r_ between the leftmost and rightmost magnetosome decreases more strongly over the course of 10 hours (Figure 2D). The fast increase in magnetization reflects the high probability that these simulations lead to the formation of a single magnetosome chain, as in the example shown in Figure 3B. Note that, in this example, the chain is slightly off-set from the center of the cell, indicating that growth from the nucleated chain occurs randomly in both directions with no mechanism for correcting such asymmetries. Some magnetosomes can be seen to be far away from the chain in the cell center, diffusing (almost) freely though the cell. We note that these magnetosomes interact with the chain magnetically, as their magnetic moments are aligned with respect to the chain.

The quantitative analysis of the structures formed after 10 hours shows that these observations are typical. For low magnetosome mobility, the results are almost the same as in the absence of such binding sites. With increasing magnetosome mobility (*D*>100 nm^2^/s) however, we see a faster decrease of the number of chains per cell in the presence of binding sites than in their absence (Figure 4A, dashed green and solid black lines, respectively), a stronger increase in the fraction of cells that have a single chain (Figure 4B), and a stronger decrease in the fraction of cells with chains of opposite polarity (Figure 4C). These observations depend on the length of the binding zone; when the lengths of the binding zone is reduced to *L*_b_ = 500 nm, corresponding to binding sites for 10 closely packed magnetosomes, the presence or absence of these binding sites made no difference for the formation of a single chain (compare the solid green line with the black lines in Figure 4A–C), indicating a rather strict requirements for the robust functioning of such mechanism. However, both the short and long binding zones concentrated the magnetosomes towards the center of the cell, as indicated by the position of their center of mass (Figure 4D).

### Case study 3: magnetism on and active movement on

Another possibility is that active transport towards the center of the cell is responsible for chain assembly as well as for the positioning of the chain. Such a mechanism might for example be driven by the polymerization or depolymerization of cytoskeletal filaments [33]. In magnetotactic bacteria, filaments formed by MamK [13], [23], the FtsZ-like protein [34] and a MamK-like protein [24] are candidates for such polymerization motors (discussed below). If the case without active movements describes the *mamK* deletion mutant (see the Discussion), this case corresponds to the situation in wildtype cells. As in the case of defined binding sites for chain nucleation, such a mechanism requires that the cell can determine its center. We have implemented active transport in our simulations by a constant active force towards the cell center that acts on all magnetosomes containing a crystal (the latter assumption is discussed below). If such an active force is indeed generated by polymerization of cytoskeletal filaments, it can be expected to be of the order of a few pN.

In the presence of an active force *F*_act_ = 1 pN, the magnetization of the cell rises at an earlier time than its absence (∼2 hours after induction rather than ∼3 hours). This lag time is comparable to experimental observations, where the magnetization rises about 1.5 hours after induction [20]. For low magnetosome mobility, it also rises about 2-fold faster (compare the black and red lines in Figure 2C). An even more pronounced effect is the fast decrease of *d*_l-r_, the average distance between the leftmost and rightmost crystal (Figure 2D). *d*_l-r_ also gets much closer to its minimal possible value (1450 nm for close packing of 30 magnetosomes), which corresponds to close packing of the magnetosomes in a single chain, than in simulations with only magnetic interactions and diffusion or with a binding zone.

Figure 3C shows an example of a simulation with an active force of 1 pN. Essentially all magnetosomes that have nucleated a crystal are found in the center of the cell, in contrast to the case with a binding zone shown in Figure 3B. Initially both magnetic orientations coexist, but as the crystals keep growing, the magnetic interactions become stronger and force the magnetosomes to align in one chain. The chain as a whole is rather immobile in the center of the cell, but individual magnetosomes from both ends of the chain can be seen to make diffusive excursions away from the chain. For a lower active force, these diffusive excursions are bigger, and we also observe diffusive movements of the whole chain (Figure 3D, *F*_act_ = 0.01 pN).

We have analyzed the structures formed after 10 hours in the same way as before. The results are shown in Figure 4 for three values of the active force (*F*_act_ = 0.01, 0.1, and 1 pN). For sufficiently large magnetosome mobility, the formation of a single magnetosome chain is very robust: For active forces of 1 pN and 0.1 pN, all simulations with *D*>10 nm^2^/s form a single chain whereas for a force of 0.01 pN a small fraction of cells forms more than one chain, even for very mobile magnetosomes. For low magnetosome mobility, however, the presence of an active force has only a small effect. The transition between the two regimes is characterized by a rather sharp threshold mobility above which the dynamics is dominated by the active forces (Figure 4A–C). The threshold is shifted to lower values for larger active forces (*D*>100 nm^2^/s for 0.01 pN, >10 nm^2^/s for 0.1 pN, and >1 nm^2^/s for 1 pN). The anticorrelation of the required mobility and the driving force indicates that reliable chain formation requires a minimal magnetosome velocity, which can be estimated as *v*_min_ = *DF*_act_/(*kT*)≈15 nm/min. The latter value is easily accessible for polymerization motors, which can generate velocities of the order of ∼1 µm/min [35], [36]. The requirement for a minimal velocity indicates that the transport of magnetosome to the assembling chain in the cell center competes with another process; and an inspection of the trajectories of simulated magnetosomes suggests that this competing process is the assembly of short chains at off-center positions. Such chains may interfere with the assembly of the centered chain in at least two ways: (i) by slowing down the dynamics, because once such chains have formed, joining them requires either the disassembly of a chain or its transport, which is slower than the transport of individual magnetosomes; or (ii) by developing independent magnetic moments in the short chains with magnetic repulsion between them. Simulations with modified magnetic interactions show no change in the threshold mobility (Figure S1), suggesting that the slow-down of the dynamics is the main dynamical barrier arising from the formation of several chains.

The presence of an active force decreases the average number of chains per cell (Figure 4A) and increases the probability that a cell forms a single chain (Figure 4B) compared to both the case with only magnetic forces and the case of a binding zone for chain nucleation. It also results in better-centered chains than both other models (Figure 4D). We therefore conclude that active transport can account for both the formation of a single magnetosome chain and its central positioning.

**Figure 5:**
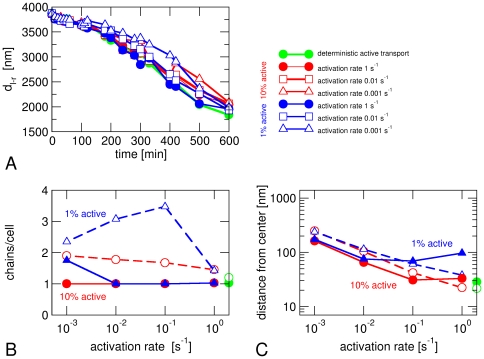
Stochastic active transport. Data from simulations where active transport of a magnetosomes is switched on and off stochastically. Transport is on during 10% (red) or 1% of the time (blue) with different activation rates. (A) Time evolution of the distance *d*_l-r_ between the leftmost and rightmost magnetosome. (B) Average number of chains per cell and (C) distance of the magnetosome center of mass from the cell center after 10 hours. Green circles indicate the results for active transport that is constitutively on. Open symbols indicate the corresponding results in the absence of magnetic interactions. All data shown here are from simulations with *D* = 1000 nm^2^/s and an average active force of 0.1 pN.

We also implemented a stochastic version of active transport in which each magnetosome switches stochastically between states in which it is transported actively or not (Figure 5). These random switching events may for example represent binding and unbinding to/from cytoskeletal filaments by which or along which bound magnetosomes move, as has been studied for eukaryotic cytoskeletal transport [37]. The effect of stochasticity on chain formation is small (Figure 5A and 5B), but centering of the chains becomes less accurate (Figure 5C). This reduction in centering reflects the increased ability of magnetosomes to diffuse away from the center when active transport is off.

**Figure 6:**
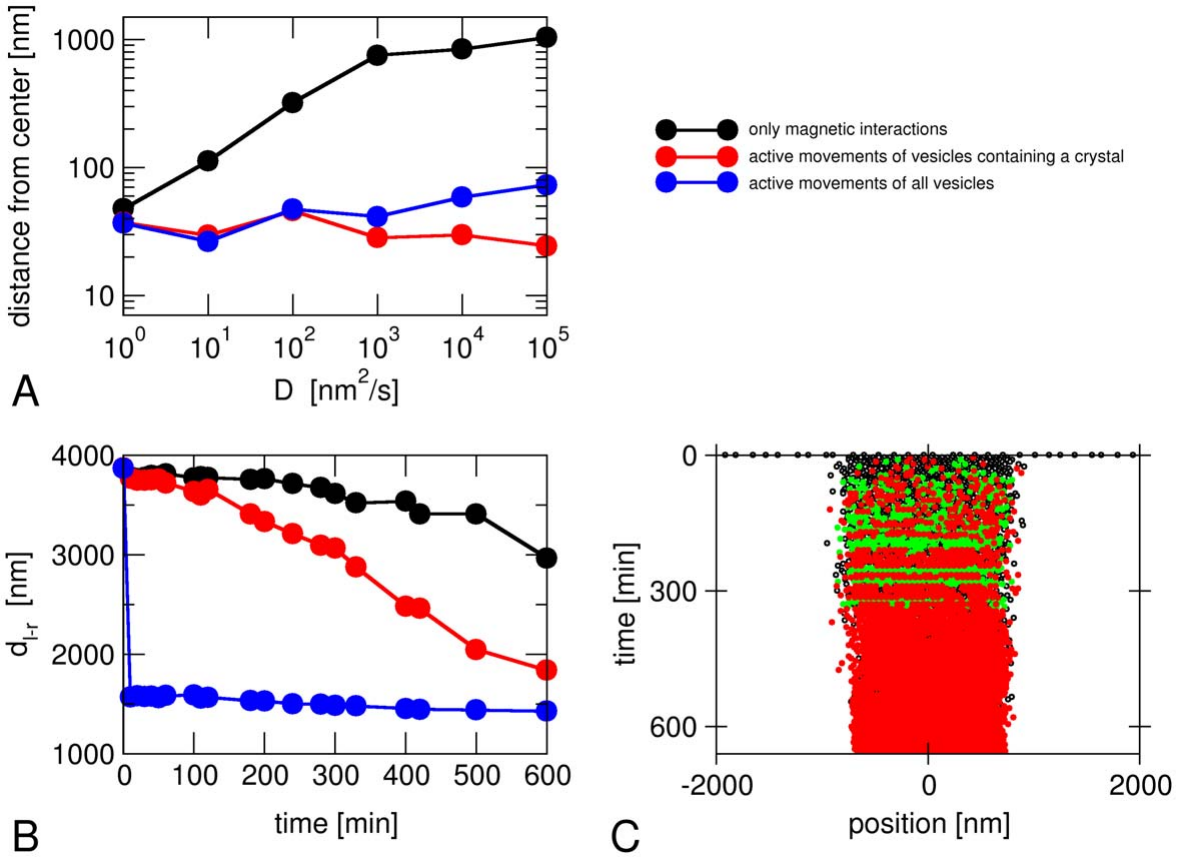
Simulations with active transport of empty vesicles. (A) Average distance of the magnetosome chain from the center of the cell after 10 hours for cases with active transport of all vesicles (blue) or only those containing a crystal (red). The case without active transport (black) is also shown for comparison. (B) Distance *d*_l-r_ between the leftmost and rightmost magnetosome vesicle as a function of time, (C) Time course of the magnetosome positions (as in Figure 3) for a case with active transport of all vesicles. The parameters are *D* = 1000 nm^2^/s, *F*_act_ = 0.1 pN.

In the simulations described so far, only magnetosomes that contain a nucleated crystal were subject to active transport. This assumption is based on the idea that formation of the mineral in the magnetosome vesicle and transport towards the cell center are activated together. These simulations reproduce the observed behavior of *M. gryphiswaldense*, where nucleation of crystals occurs throughout the whole cell before the vesicles are assembled in a chain [14], [20]. By contrast, we observed that chains of empty vesicles form before crystals are nucleated when we implemented active movements for all vesicles, independent of whether they contain a crystal (Figure 6). This is a phenotype typical of *M. magneticum* where chains of empty vesicles have been observed [38]. These observations have been proposed to reflect inherent differences between the two closely related species [39]. Thus, an additional result of our simulations is that this important mechanistic difference between the two strains is linked to the activation of magnetosome movements with or without activation of biomineralization.

### Case study 4: magnetism off and active transport on

Finally, we have simulated crystals without a magnetic moment, but subject to active transport to test what role the magnetic interactions have in the process. This type of ‘mutation’ is extremely unlikely to be realized experimentally as a mutant forming non-magnetic mineral would have to be generated. The results for the average distance between the leftmost and rightmost magnetosome (Figure 2D), the number of chains per cell (Figure 4A) and the centering of the magnetosome chain (Figure 4D) are very similar as in the presence of magnetic interactions, but for small active forces, the number of chains per cell is slightly increased in the absence of magnetic interactions and the probability of finding only one chain is reduced by approximately 25% (dashed red and blue lines in Figure 4A and 4B). These effects become much more pronounced in the stochastic model of active transport (Figure 5B). We interpret these observations as reflecting the lower cohesiveness of chains, as the magnetosomes become more mobile and can more easily make diffusive excursions away from the chain. This result is in agreement with observations from FMR spectroscopy that also suggested a role for magnetic interactions between magnetosomes for the stabilization of the chain [20]. Our simulation results thus suggest that attractive magnetic interactions are not the main driving force for the formation of the magnetosome chain, but play a role in stabilizing the chain, after active transport has brought magnetosomes in close proximity.

## Discussion

We have implemented a computer model to study the interplay of transport processes and physical interactions for the assembly of magnetosome chains in a bacterial cell. The simulations show that diffusive movements of magnetosomes, guided by their magnetic interactions, are not sufficient for the robust formation of a single magnetosome chain that is observed experimentally in magnetotactic bacteria. Our results also indicate that cytoskeletal structures such as the MamK filaments play a key role in the assembly of the magnetosome chain beyond constraining the magnetosomes into a linear arrangement and stabilizing it against collapse into unstructured clusters [13], [14], in agreement with recent observations on a *mamK* mutant (discussed below). We have considered two mechanisms that allow for better control of the chain's assembly, both of which rely on a cytoskeletal scaffold at the cell center or directed towards it: a binding zone that nucleates the assembly of a magnetosome chain and active transport towards the cell center. Both mechanisms perform better than simple diffusion and magnetic forces to assemble a single magnetosome chain and to localize it in the cell center. So far, both mechanisms are hypothetical and it is not clear which one is realized in bacterial cells. We currently favor an active transport mechanism, based on the following reasoning: (i) it is known that magnetosome filaments are dynamic [23], which suggests that they are good candidates for polymerization motors; furthermore, active transport by polymerization motors has been demonstrated to occur in bacterial cells, driving for example plasmid segregation [40]; (ii) the magnitude of the active forces required for reliable chain assembly in our simulations is easily in the range that can be achieved by such polymerization motors (see below); (iii) by contrast, although a binding zone can nucleate a single magnetosome chain in our simulations, this mechanism needs to fulfill relatively strong requirements in order to do so; in particular a small nucleus is not sufficient (Figure 4). We also want to emphasize that, despite the central role of active transport in the scenario suggested by our simulations, the magnetic interactions between magnetosomes also play an important role by providing additional stability to the chain, in particular in the stochastic version of our model for active magnetosome movements.

Within our model, the assembly of the magnetosome chain is very reliable if the value of the active force exceeds about 0.1 pN, provided that the transport mechanism is constitutively active. Forces of this order of magnitude are well within the range that can be generated by cytoskeletal molecular motors or by the active polymerization of cytoskeletal filaments: A single polymerizing microtubule can generate a force of 3–4 pN [35] and bundles of actin filaments generate about 1 pN [41]. Both actin- and tubulin-related force-generating proteins have been identified in bacteria, for example the actin-like MreB and ParM proteins that drive DNA segregation [40], [42] and the tubulin-related FtsZ believed to drive constriction of the cell division septum [31]. A key question that remains open is which molecular players are involved in the generation of active magnetosome movements. Specific candidates for active force generation in magnetotactic bacteria are MamK [13], [23] (which is very likely to have an important role as discussed below) and a MamK-like protein [24], which are actin-related, and an FtsZ-like protein [34]. All three proteins form filaments in a nucleotide-dependent manner. Moreover, further cytoskeletal proteins may be discovered as the genomes of magnetotactic bacteria have only very recently started to be studied [43].

**Figure 7:**
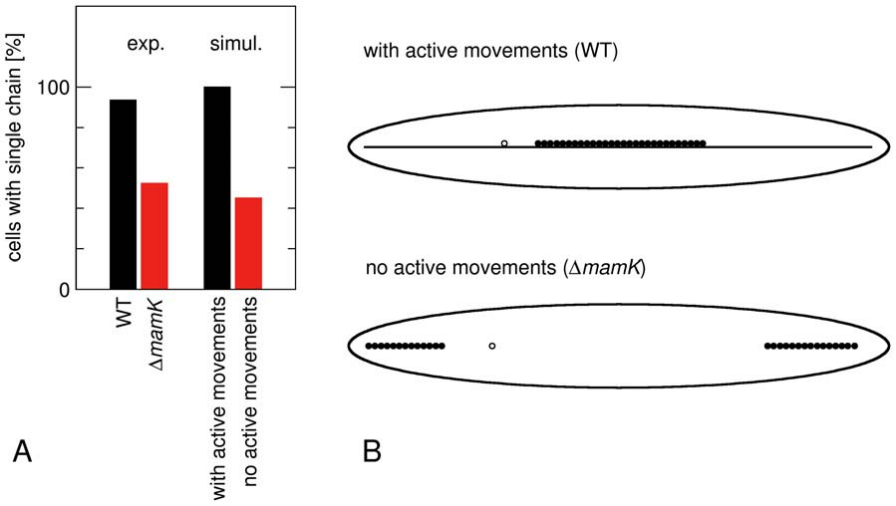
Comparison of chain formation in simulations and experiments. (A) Fractions of cells with a single chain as observed from electron microscopy images of *M. gryphiswaldense* wild type and Δ*mamK* cells [15] and from our simulations with and without active movements of magnetosomes (data from Fig. 4B, with *D* = 10^4^ nm^2^/s and *F*_act_ = 0.1 pN). (B) Examples of magnetosome structures formed in these simulations. These structures are very similar to those seen in electron microscopy images of Ref. [15].

Remarkably, the structures we find in our simulations of magnetosome dynamics without active movements (black circles in Figure 4, see also Figure 7) strongly resemble recent observations of a *mamK* deletion mutant in *M. gryphiswaldense* [15] (similar observations have also been made for *M. magneticum* [13]). Cells of this strain do not form MamK filaments, and have a smaller numbers of magnetosomes. Most interestingly, however, formation of magnetosome chains is observed despite the absence of MamK, but about half of the cells contain 2–4 short chains rather than one long chain (Figure 7), and chains are displaced from midcell [15]. Our model thus suggests an interpretation of the *mamK* phenotype as the loss of or a defect in the active transport of magnetosomes towards the cell center. In that case, short chains may form in multiple locations in the cell. These short chains move too slowly to merge into a single chain by diffusion and build up independent magnetic moments, which may provide additional barriers for the concatenation of two chains if they have opposite orientation. We note that, while these observations point to a crucial role of MamK for the active movements of magnetosomes, the mechanistic role of MamK remains an open question. MamK is a candidate for the motor protein that generates force and drives active magnetosome movements, but its may also have some other function that is required for these movements.

Our simulations also indicate that a coupling between the activation of biomineralization in a vesicle and the activation of its active transport is required in order to reproduce the dynamics of chain formation that is observed experimentally in *M. gryphiswaldense*, where crystals are nucleated in magnetosome vesicles throughout the cell before a chain of magnetosomes is formed [14], [20]. Without such coupling, our simulations rapidly form chains of empty magnetosome vesicles as observed in *M. magneticum* [38], but not *M. gryphiswaldense* [14], [20]. The simulations therefore suggest that an important mechanistic difference between these related species is based on the presence or absence of such coupling of the activation of magnetosome mobility and the activation of biomineralization.

In summary, our simulations suggest that active movement is the main driving force for the formation of a magnetosome chain. We have focused on the de novo formation of such a chain in iron-starved non-growing cells, a situation that has been addressed in numerous experimental studies [14], [15], [17], [18], [20]. However, active transport of magnetosomes is a general mechanism that could also explain the growth of magnetosomes in cells that grow and proliferate as well as the movement of a chain to the cell center after cell division [44]. Both cases can be studied with the model proposed here and experiments addressing their dynamics may provide additional constraints on the remaining unknown parameters, in particular the magnetosome mobility. In a wider context, our results also point toward a direction for the formation of one-dimensional magnetic inorganic-organic nanostructures and their possible applications in bio- and nanotechnologies [45]. The assembly and manipulation of magnetic chains indeed remains a challenge, and further scientific and technological advances in their application rely on the ability to organize them into controllable, ordered, and hierarchical structures.

## Materials and Methods

### Simulation of magnetosome movements

The spatial degrees of freedom of the magnetosomes, independent of whether or not they contain magnetite crystals, are modeled as one-dimensional movements to mimic their arrangement along magnetosome filaments. These movements are described by a set of Langevin equations for the magnetosome positions *x_i_*,
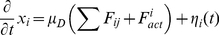
(1)where *F_ij_* describes the interactions between magnetosomes, *F^i^_act_* is a force arising from active transport processes and h is a white noise term that describes the source of diffusive movements of the magnetosomes. The latter are described by the magnetosome diffusion coefficient *D*, which also defines the mobility coefficient μ_D_ through μ_D_ = *D/kT* with *T* taken to be room temperature (300 K). Magnetosomes interact through magnetic dipole-dipole interactions, which can be either attractive or repulsive, and short-range hard-core repulsion. The magnetic interactions of two magnetosomes are characterized by the force 
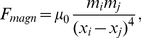
(2)which is directed towards the center of mass of the two particles. Here *m_i_* and *m_j_* are the magnetic moments of the two magnetosomes and μ_0_ = 4π×10^−7^ N/A^2^. To account for the short-range repulsion between magnetosomes, we only allow them to overlap over a distance d. Larger overlap between particles is prevented by a hard wall potential at a distance between consecutive magnetosomes of 2×*R*_max_−d. If an integration step in our simulations would lead to such overlap, magnetosomes are only moved as far as it is possible without creating such overlap. As this implementation of a hard wall does not have a well-defined equilibrium position, where the force between two magnetosomes vanishes, we also introduce the following modification for attractive magnetic interactions at short distances: We take the force to be given by Eq. (2) if the distance between two magnetosomes is larger than 2*R*_max_+d. For distances between 2*R*_max_ and 2R_max_+d, the absolute value of this force is taken to be linear in the distance such that it vanishes for a distance 2*R*_max_ and reaches the value given by Eq. (2) for a distance 2*R*_max_+d. For distances between 2*R*_max_ and 2*R*_max_−d, we introduce a very weak repulsive force that is also linear in the distance and has the absolute value *F*_rep_ for a distance 2*R*_max_−d and vanishes at 2*R*_max_. Finally, if, without hard wall implementation, an integration step would have resulted in overlapping magnetosomes and one of those is an empty vesicle, we allow the two magnetosomes to exchange their positions with probability *p*_sw_. This additional move accounts for the fact that magnetosome movements in the cell are not strictly one-dimensional and that two magnetosomes may pass each other while both are connected to a cytoskeletal structure, but on opposite sides, and prevents empty vesicles to act as barriers for chain formation. As the precise microscopic interactions between magnetosomes are not known, our implementation of the short-range interactions does not aim at a realistic description of the system, but rather has been chosen to implement generic aspects of such interactions, while at the same time allowing for simulations with a relatively large time step, so that we can run simulations of the magnetosome dynamics over the experimental time scale of several hours.

### Simulation of crystal growth

In our simulations, the number (*N*) and size (*R*_max_) of magnetosome vesicles is fixed.

As we focus on the de novo formation of magnetosome chains in non-growing cells after a shift to iron-containing medium [14], [15], [17], [18], [20], [29], we start the simulation with *N* = 30 empty magnetosome vesicles, distributed randomly in a cell, and simulate the time evolution of crystal growth and magnetosome movements over 10 or 11 hours.

Magnetite crystals are nucleated stochastically in empty magnetosome vesicles with rate ν. Once nucleation has occurred, the growth of the crystal is deterministic and the crystal volume increases linearly in time, *V*_i_ = *v*_gr_ (*t−t_i_*), where *t_i_* is the time of nucleation in magnetosome *i*, until the maximal size, defined by the maximal radius *R*_max_ is reached. In every simulation step we therefore allow each empty magnetosome vesicles to nucleate a crystal with probability νΔt, and every existing crystal smaller than the maximal size is increased by *v*_gr_Δt.

### Simulation of the magnetic moments

The magnetic moment of a magnetosome is described by a single spin-like variable, i.e. a magnetic moment along the axis of the cell that can attain the values *m_i_* = ±μ*V_i_* with μ = 6×10^5^ pN/A^2^, when the magnetosome crystal has volume *V_i_*. The absolute value of the magnetic moment of a crystal increases deterministically with time as the crystal grows, while its orientation evolves according to a Monte Carlo method. At every time step of the simulation, we perform a Monte Carlo move for every crystal. If the crystal is in the superparamagnetic state, which is attained for crystals smaller than a critical radius *R*_crit_ [46], we flip its spin with Metropolis rates [47] that are calculated from the energy of the magnetic moment of that crystal in the magnetic field that arises from the magnetic moments of all other crystals. As we perform such Monte Carlo moves at every time step, the magnetic degrees of freedom of small crystals are effectively equilibrated on the time scale of the spatial dynamics of the magnetosomes. In the results presented in Figure 2C, we have therefore averaged the instantaneous values of the magnetization over time interval of 5 min. Magnetite crystals larger than the critical radius *R*_crit_ are in the stable single domain state and exhibit hysteresis [46]. In this case, a magnetic field exceeding the coercive field *B*_coerc_ is required to reverse the magnetization [46]. In our simulations, hysteresis is implemented in the following way: If the crystal is larger than *R*_crit_, the magnetic field is calculated as for the calculation of the Metropolis rates, but a spin flip is only performed if the field is opposite to the current magnetization and its absolute value exceeds *B*_coerc_.

## Supporting Information

Figure S1 **Simulations with modified magnetic interactions.** (A) Number of chains per cell and (B) fraction of cells with a single chain from simulations where the coercive field has been increased (blue) or the repulsive part of the magnetic dipole-dipole interactions has been omitted (green).
(TIF)
