# Supplementary figures and images for: Interplay of Magnetic Interactions and Active Movements in the Formation of Magnetosome Chains
Source: PLoS One. 2012 Mar 19;7(3):e33562. doi: 10.1371/journal.pone.0033562 (PMC3307741; doi:10.1371/journal.pone.0033562)

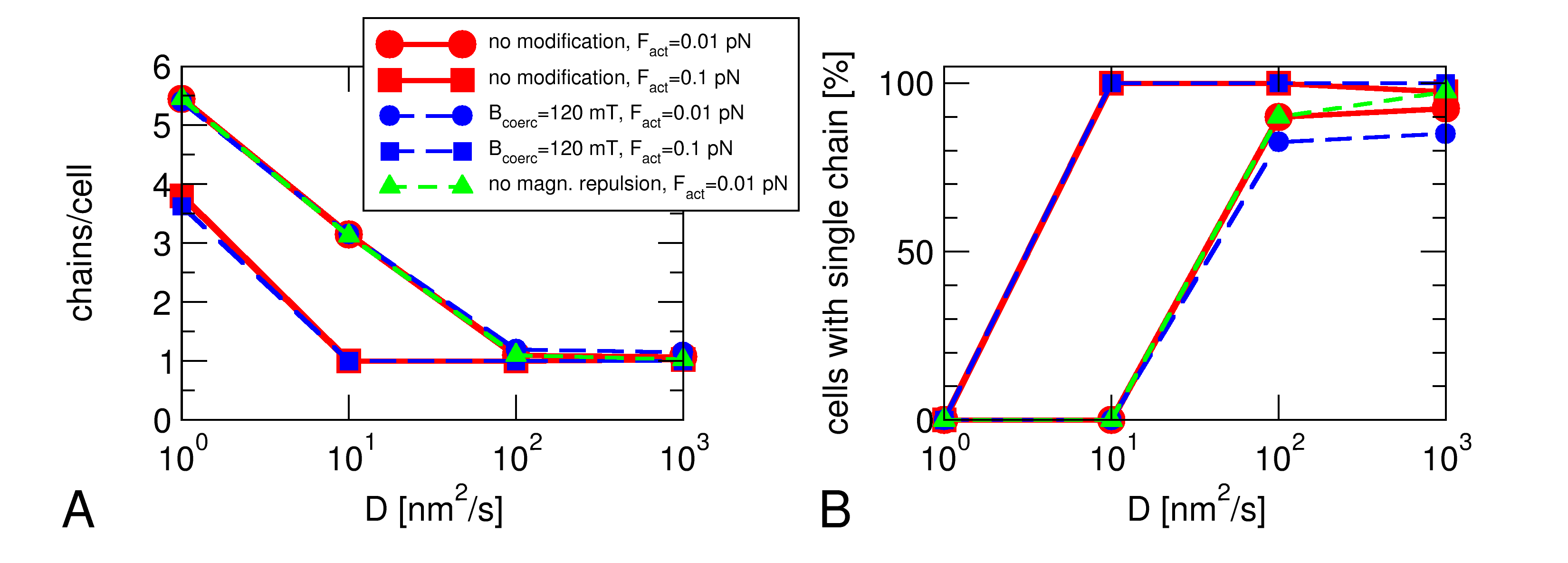

Supplement: Figure S1 — Simulations with modified magnetic interactions. (A) Number of chains per cell and (B) fraction of cells with a single chain from simulations where the coercive field has been increased (blue) or the repulsive part of the magnetic dipole-dipole interactions has been omitted (green). (TIF) [file pone.0033562.s001.tif]
